# The Icarus Flight of Perinatal Stem and Renal Progenitor Cells Within Immune System

**DOI:** 10.3389/fimmu.2022.840146

**Published:** 2022-03-09

**Authors:** Angela Picerno, Giuseppe Castellano, Claudia Curci, Katarzyna Kopaczka, Alessandra Stasi, Giovanni Battista Pertosa, Carlo Sabbà, Loreto Gesualdo, Roberto Gramignoli, Fabio Sallustio

**Affiliations:** ^1^ Department of Interdisciplinary Medicine, University of Bari “Aldo Moro”, Bari, Italy; ^2^ Nephrology, Dialysis and Renal Transplant Unit, Department of Clinical Sciences and Community Health, University of Milan, Milan, Italy; ^3^ Nephrology, Dialysis and Transplantation Unit, Department of Emergency and Organ Transplantation (DETO), University of Bari Aldo Moro, Bari, Italy; ^4^ Department of Laboratory Medicine, Division of Pathology, Karolinska Institutet, Stockholm, Sweden

**Keywords:** immune system, stem cells, renal stem cells, perinatal stem cells, immunomodulation, T cells, inflammatory response

## Abstract

Our immune system actively fights bacteria and viruses, and it must strike a delicate balance between over- and under-reaction, just like Daedalus and Icarus in Greek mythology, who could not escape their imprisonment by flying too high or too low. Both human amniotic epithelial and mesenchymal stromal cells and the conditioned medium generated from their culture exert multiple immunosuppressive activities. They have strong immunomodulatory properties that are influenced by the types and intensity of inflammatory stimuli present in the microenvironment. Notably, very recently, the immunomodulatory activity of human adult renal stem/progenitor cells (ARPCs) has been discovered. ARPCs cause a decrease in Tregs and CD3^+^ CD4^−^ CD8^−^ (DN) T cells in the early stages of inflammation, encouraging inflammation, and an increase in the late stages of inflammation, favoring inflammation quenching. If the inflammatory trigger continues, however, ARPCs cause a further increase in DN T cells to avoid the development of a harmful inflammatory state. As in the flight of Daedalus and Icarus, who could not fly too high or too low to not destroy their wings by the heat of the sun or the humidity of the sea, in response to an inflammatory environment, stem cells seem to behave by paying attention to regulating T cells in the balance between immune tolerance and autoimmunity. Recognizing the existence of both suppressive and stimulatory properties, and the mechanisms that underpin the duality of immune reaction, will aid in the development of active immunotherapeutic approaches that manipulate the immune system to achieve therapeutic benefit.

## Immune Response in Homeostasis or Healing Process

Human beings are regularly exposed to millions of potential pathogen events and organisms, through contact, inhalation or ingestion. Our innate ability to prevent or respond to pathogens is not specific, the adaptive immune response is. Innate immune response is common among vertebrates and invertebrates, and also plants, shielding and preserving them. Approximately 500 million years ago, we have pieces of evidence supporting the rise of adaptive immune system in the evolution, confinely to vertebrates, in response to complex systems and growing risks associated with elaborate activities and metabolism. The adaptive immune response is slow, particularly for the first antigen encounter. It may take days when not weeks to activate and expand specific clones of B and T cells, and we rely on such long-term memory to react against previous encountered pathogens (1).

Both innate and adaptive immune components are daily involved in preventing bacterial and viral infections, striking a delicate balance between over- and under-reaction to such stimuli. Such delicate and strict activity bears resemblance to the Greek myth of “Daedalus and Icarus”, two mythological figures whose restrained and regulated flight, neither too high nor too low, led to successful imprisonment break. At the same manner, when the human immune system is out of balance, not only does it fail to defend the body, but it can even over-react attacking itself. A deranged, rampant immune response may mistake “self” cells for invading pathogens, leading to devastating auto-immune disorders, like lupus and rheumatoid arthritis. At the same manner, when the immune system misidentifies a harmless particle (such as pollen) as an invading parasite, allergies can develop and escalate to life-threating disease. For a long time, immunologists and cell biologists have been struggling to identify molecular pathways involved in such balance and constrain or maneuver immune response towards favorable counteroffensive.

In addition to infective prevention, the innate and adaptive immune response maintains homeostasis and supports innate healing process. Homeostasis is the essential process aimed to preserve normal, healthy ranges for critical factors as energy intake, body temperature and growth. Such articulated and entangled system has the well-known ability to react to external stimuli. In the “Cellular Immunology” book, Barnet offered an important reflection on clonal selection hypothesis, suggesting as “self-defense” was originally concerned not with defense against infection but with the preservation of cellular integrity of the body (2).

When a body is under attack, infected by micro-organisms, it needs to invest a lot of energy to fight invaders back, through immune cellular response. Nature developed an intriguing mechanism, privileging immune reaction over homeostasis (such as preserving hydration or temperature levels), in order to ensure survival of the whole body in such critical event. Nevertheless, homeostasis needs to be reassured as soon as possible, and immune response is suddenly restored once the insult is contained and the risk removed.

Therefore, scientists immediately recognized the importance in understanding the molecular aspects of such critical response, and they have been making huge efforts to understand and develop a method to control and eventually enhance (innate and/or adaptive) immune system.

In this delicate balance also some types of stem cells play a role. Mesenchymal stromal cells (MSC) can move towards inflammatory areas and exert immunomodulatory and anti-inflammatory effects *via* cell-to-cell contacts with lymphocytes or *via* the generation of bioactive molecules, such as cytokines, growth factors, and chemokines, that have autocrine/paracrine effects (3). During the past decades, embryonic and perinatal stem cells (derived from extra-embryonal and gestational tissues) have attracted interest and endeavor. In particular, perinatal cells have been recently gained recognition as advanced therapy medical products proposed in regenerative applications (4).

Recently, it has been discovered that also human adult renal stem/progenitor cells (ARPC) have a potent immunomodulatory capacity and may contribute locally to limit tissue damage and inflammation (5). Researchers are considering new ways to treat autoimmune disorders, sepsis, and transplant surgery, exploiting these cells (6).

## Human Amniotic Epithelial Cells and Their Immune-Modulatory Molecules

Placenta-derived perinatal stem cells are characterized by steady immunomodulatory capacity to nourish protection from maternal immune system. During a 9-month incubation, the (semi)allogeneic fetus requires maternal immune adaption and acquisition of tolerance at the maternal–fetal interface. Although decidua-resident T cells and macrophages, characterized by regulatory properties, have been recently described playing a central role in promoting fetal tolerance, stromal cells have been largely implicated in modulatory and tolerogenic effects (7). The inner layer of human placenta, amnion-chorionic layer and amnion membrane in particular, represents a protective barrier against maternal recognition and fetal rejection. Several groups have recently collected and described evidence in support of restraining effects granted by human amniotic epithelial cells (hAEC) towards immune effector cells. Recent reports describe altered maturation of antigen-presenting or dendritic cells, inhibitory effect on natural killer (NK) cell, decreased circulatory mononuclear cell proliferation or activation, and switch toward regulatory phenotype in B and T cells or macrophages (8–11). All these immune-regulatory properties, rather than immune-suppressive effects, have successfully bridged the embryo during a 9-month development and may convey towards new, innovative cellular treatments for regenerative purposes.

Embryonic and perinatal tissues possess potent immune-protective properties, not fully understood or characterized. Constitutive expression of surface proteins and enzymes, couple with secreted forms of modulatory mediators have been recently described and reported critical in modulatory processes. Initially, peculiar expression of tolerogenic human leukocyte antigen G (HLA-G), both at mRNA and protein levels, has been reported in embryonic tissues (12, 13), and lately identified and described in fetal and maternal layers of human placenta (11, 14). Conversely, to ubiquitously expressed polymorphic HLA class Ia antigens, HLA-1b molecules display a restricted pattern of expression in selected tissues (as thymus, cornea, proximal nail matrix and erythroblasts) in healthy individuals (15–17). HLA-G protein was controversy described also in MSC (18), lately restricted to soluble forms (19). Soluble form of HLA-G (sHLA-G) has been measured in the serum or plasma, secreted by monocytes or T cells in pathological conditions, such as inflammatory diseases or viral infections, or in neoplastic and autoimmune disorders, but also in response to solid organ transplantation (20, 21).

On the contrary, trophoblast cells and epithelial cells lining the umbilical cord or amnion membrane have recently described to expressed both membrane-bound molecules and release soluble forms of HLA-G and HLA-E (both HLA-1b molecules) (14, 22, 23). The non-polymorphic HLA-1b expression has been correlated to other paracrine factors (i.e., chemo- or cyto-kines as IFN-gamma, TGF-beta, IDO, GM-CSF, pro-inflammatory or anti-inflammatory interleukins) (24–27) or to oxygen tension (hypoxic or near-hypoxic conditions) (28).

**Figure 1:**
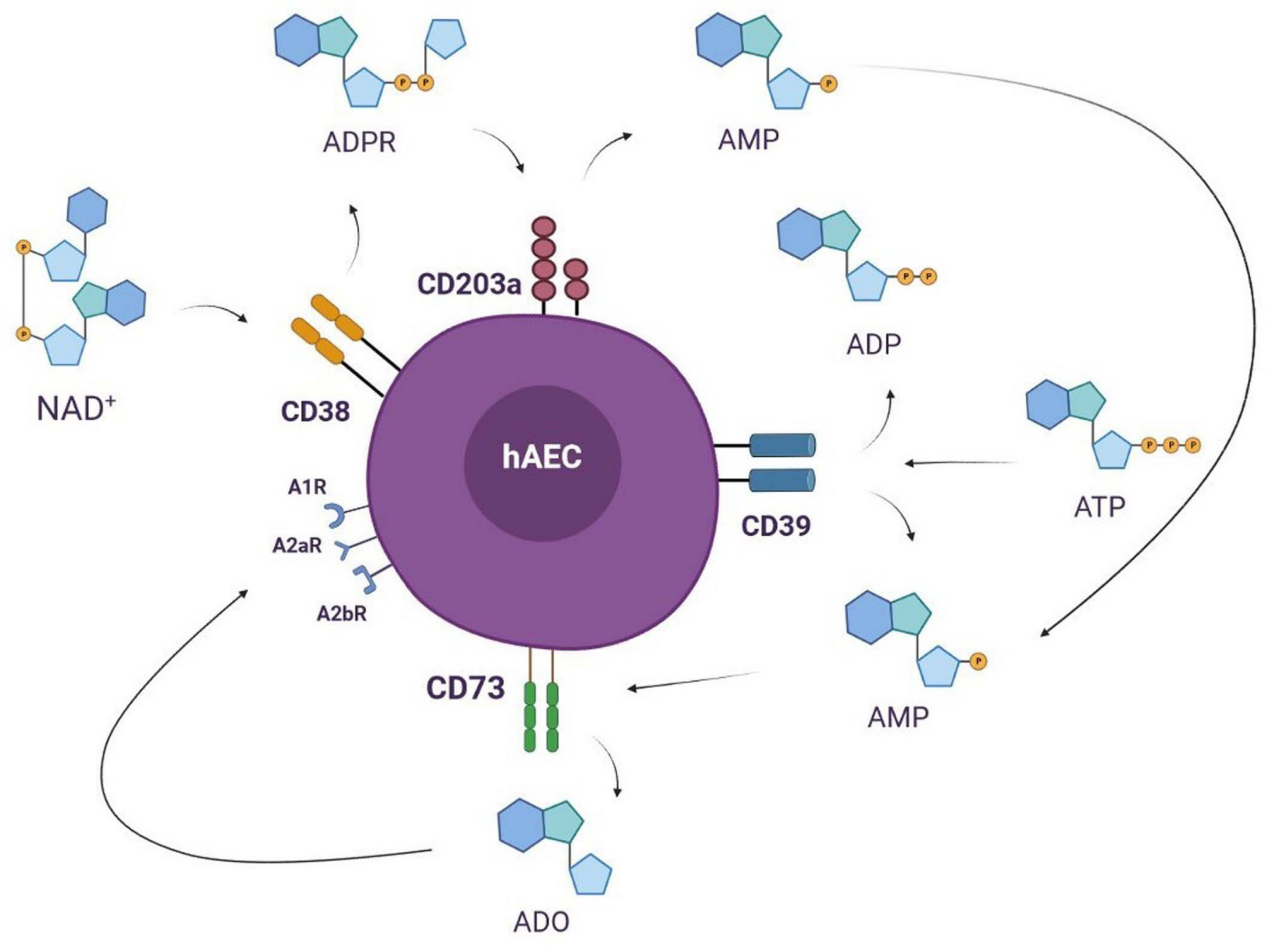
The canonical pathway of ADO production initiates with CD39, an ecto- diphosphohydrolase responsible for hydrolyzation into mono- or di-phosphate nucleotide which are suddenly converted into soluble ADO by CD73. CD39 and CD73 can be present on the same cell or in close proximity when two cellular elements are adjacent. Interestingly, hAEC has been recently proved to constitutively expressed both canonical ecto-enzymes on membrane surface. However, such “canonical” way is not the sole molecular pathway responsible extracellular ADO production. Another “alternative” pathway has been described where nicotinamide adenine dinucleotide (NAD^+^), rather than ATP, is the initiating factor for ADO production (and relative immune cell modulation). NAD can be actively secreted across cell membrane and trigger a cascade of extracellular signals, starting with CD38 resulting in soluble ADPR. CD38, another membrane ecto-nucleotidase, lends ADPR over to CD203a, a surface nucleotide pyrophosphatase/phosphodiesterase 1, that generate mono-phosphate adenosine (AMP). Similarly to “classical” pathway, AMP generated in the “alternative” cascade is converted into ADO by CD73, the point of contact for the two canonical and alternative pathways.

Among immunomodulatory molecules, adenosine (ADO) has recently gained an important role in different patho-physiological settings and in innovative cell-based therapies. Extracellular ATP is the primary substrate for ADO typically hydrolyzed by membrane-bound ectoenzymes as CD73 (5′-nucleotidase). CD73, even if expressed by several cells, has been for long time recognized as identity marker for an efficient and rapid identification of MSC products (29). CD73 represents the final ecto-enzyme to complete the adenosinergic loop (30). A membrane-bound ecto-nucleoside hydrolase (CD39), present on the same cell or another adjacent element, is responsible for hydrolyzation into mono- or di-phosphate nucleotide offered to CD73 in the so called “canonical” pathway (31). However, such “canonical” way is not the sole molecular pathway responsible for T cell modulation. Another “alternative” pathway has been recently described where nicotinamide adenine dinucleotide (NAD^+^) rather than ATP is the initiating factor for modulation for T lymphocytes (32). Nicotinamide adenine dinucleotide can be actively secreted across cell membrane and trigger a cascade of extracellular signals (27). CD38, another membrane ecto-nucleotidase, lends ADPR over to CD203a, a surface nucleotide pyrophosphatase/phosphodiesterase 1 that generates mono-phospate adenosine (AMP) (**Figure 1**). Similarly to “classical” pathway, AMP generated in the “alternative” cascade is the converted into ADO by CD73 (31).

Several aforementioned immunomodulatory pathways were initially identified and ascribed limitedly to MSC. In the position manuscript published in 2006 by the International Society for Cell and Gene Therapy (ISCT) experts, the ecto-nucleotidase enzyme CD73 was exclusively described on MSC, leading to an efficient identity marker to qualify stromal multipotent cells (29). The limited but constitutive expression of CD73 on human MSC has been lengthy maintained in quality assurance assays for MSC therapies. Such membrane-bound enzyme has also been detected, in combination or close proximity to all the aforementioned ectoenzymes, on different cell populations, namely, immune effector cells (33) or tumor cells (34). A complete loop achieved by interaction of different cells or eventually on a single cell has been proved to confer immune-protection. Recently, confirmation of constitutive, high-density presence of CD73 on the surface of hAEC has been reported. Interestingly, dichotomic effects has been measured and described in hAEC when exposed to immune cells: while T and NK cells are significantly inhibited in proliferation and activation by hAEC direct contact (10) or close proximity (23). B cell expansion *in vitro* has been surprisingly increased by few amnion cells in the compartment (10). Furthermore, ATP, NAD^+^ and ADO released into the extracellular space, have been recently described having an important role in the promotion of T and B regulatory cells and M2 macrophages (10). The same group reported for the first time the constitutive presence of all five plasma membrane nucleotidases on a single non-neoplastic cell (hAEC), suggested as such ecto-enzymatic activity plays an important role in addition to the non-polymorphic HLA-G mediation.

The most recent paradigm is that hAEC or perinatal stem cell in general, do not necessarily need to mature into adult somatic cell types (multipotency), but these fetal-derived stem cells can exert important immuno-modulatory activities *via* indirect paracrine mediators. Such mediators (collectively known as secretome) include soluble proteins (cytokines, chemokines, growth factors and proteases) (35) and extracellular vesicles (EVs) of micro- and nano-size (23). Notably, the expression of both membrane-bound HLA-G and soluble form of non-polymorphic HLA-1b molecules (sHLA-G and sHLA-E) in every primary hAEC effects on immune cells generated by surface molecules. To support the moiety in soluble molecules, an impressive amount of small and large size vesicles were released by full term hAEC, mediating anti-inflammatory, anti-fibrotic, anti-microbial, anti-apoptotic, pro-regenerative and immune-modulatory properties sufficient to restore normal architecture and function to damaged organs.

FAS receptor expression has also been observed at high levels in embryonic tissues (36, 37). Amniotic cells and their conditioned medium have been suggested for the treatment of chronic inflammation and immune alterations due to their broad immunosuppressive properties (38).

## Adult Renal Stem/Progenitor Cells: New Perspective for the T Cell Modulation

Notably, very recently the immunomodulatory activity of adult renal stem/progenitor cells (ARPCs) has been reported. Tissue-specific cells, expressing CD133 and CD24 antigens on their surface, have been described as progenitors of tubular cells and podocytes during human kidney formation (39–46). Renal progenitors are abundant in the kidneys at 8–9 weeks of gestation, when the kidney is largely made up of immature metanephric mesenchyme-derived structures; the percentage of progenitor cells decreases over the time, until they account for around 2% of renal cells in adult human kidneys (47).

ARPCs can help in kidney regeneration in two ways: by directly differentiating and by secreting reparative molecules. They can differentiate into epithelial, endothelial, osteogenic, and adipogenic cells, among others (43, 48, 49). ARPCs have been shown to be able to regenerate lengthy segments of renal tubules and missing podocytes in cortical nephrons after acute kidney injury (AKI) (50, 51). Furthermore, Toll-Like Receptor 2 (TLR2) ligands activate CD133^+^ renal progenitors, which can secrete reparative factors that can repair renal tubular cells damaged by chemical agents like cisplatin (46).

TLR2 can act as a damage sensor, and its activation can result in stem cell proliferation and differentiation, among other things. Following TLR2 stimulation, cytokines and inflammatory chemokines such as C-3 and MCP-1, IL-6, and IL-8 are released (43, 44, 52, 53). Renal progenitors can repair both physical and chemical damage, such as a wound in epithelial tissue caused by cisplatin, a widely used chemotherapeutic drug that can cause nephrotoxicity side effects. Following renal tubular cell injury, ARPCs released inhibin-A and decorin, which were directly involved in the cell regeneration process (43).

In addition, following LPS exposure, ARPCs have the ability to prevent endothelial dysfunction and protect the endothelium compartment, facilitating kidney healing. The antifibrotic activity of ARPCs is mediated by the release of antiseptic molecules CXCL6, SAA4, and BPIFA2 (54).

Recently, it has been reported, for the first time, that human ARPCs also possess immunomodulatory capabilities (5). Specifically, ARPCs have shown modulatory features towards CD3^+^ CD4^−^ CD8^−^ (double negative; DN) T cells and promotive capacity for regulatory T cells (Tregs). However, renal progenitors need to be triggered to achieve full potential immunomodulative effect. Toll Like Receptor 2 (TLR2) agonists, such as lipoteichoic acid (LTA), a major constituent of the protective wall in gram-positive bacteria, are required to stimulate innate immune responses to gram-positive bacteria (55), and activate ARPCs (46, 56). Activated ARPCs have been proved to inhibit peripheral blood mononuclear cell (PBMC) proliferation, limitedly to Treg and DN T cells. When ARPCs detected inflammation (by LTA binding on TLR2), Treg generation was inhibited both in the short- (5 days) and long-term (15 days) (5, 43). If ARPCs were not activated by LTA, a partial Treg inhibition was observed, limitedly to short-term effect. Such effect was reverted resulting in increased Treg generation in the longer term. Renal progenitor cells reach out immune cells through paracrine mediators. Soluble factors as PAI1, CXCL1/GRO-α, GM-CSF, MCP1, IL-6, IL-8, and MIF have been detected in ARPC secretome analysis when co-cultured with T cells. Interestingly, PBMC stimulation by combination of PAI1, CXCL1, MCP1, and GMCSF chemokines, had an immunomodulatory effect specifically on Treg and DN T cells.

The ability of stem/progenitor cells to affect Tregs is not new. MSC can also modulate T cell sub-populations (57, 58). Instead, what is novel is the evidence supporting stem/progenitor cell ability to modulate the recently discovered population of DN T cells. In both mice and humans, DN T cells have been described as powerful suppressor cells. They are antigen-specific suppressor cells and use trogocytosis to regulate T cells with the same antigen specificity. This is a distinguishing trait that makes them intriguing for cellular treatment, transplantation, and autoimmunity. Moreover, to control tissue immune response, proliferative DN T cells released potent anti-inflammatory cytokines such as IL-27 and IL-10 (59).

Allograft rejection, GVHD, and auto-immune diabetes can all be prevented or reversed by DN T and Treg cells (60). Their homeostatic role is attained by suppressing excessive host immune responses (61, 62). Furthermore, recent reports suggest that DN T cells in turn can regulate B cells, DCs, and NK cells (60).

**Figure 2:**
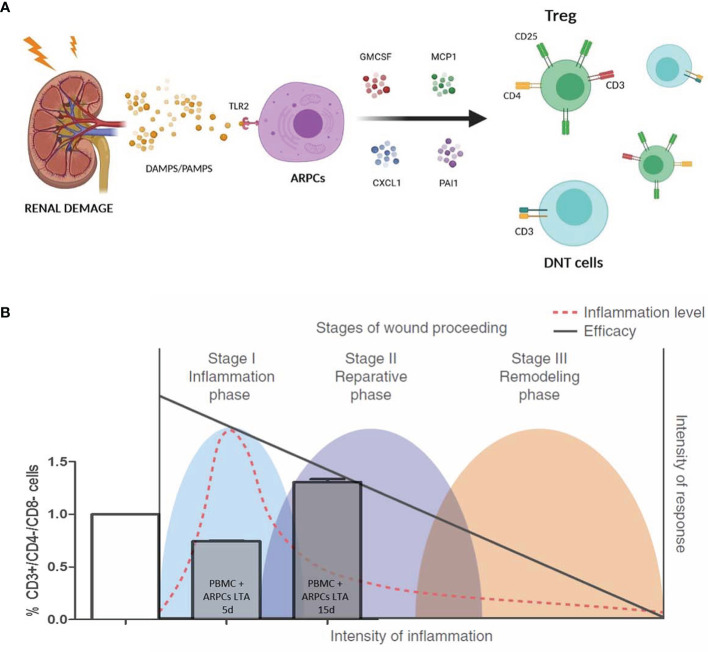
**(A)** Following a renal damage ARPCs detect the insult by the binding of Pathogen Associated Molecular Patterns (PAMPs), such as lipoteichoic acid (LTA), or damage-associated molecular patterns (DAMPs) on the Toll-like receptor 2 (TLR2). ARPCs then inhibhit the Treg and the double negative (DN) T cells through paracrine mediators as PAI1, CXCL1, GM-CSF, and MCP1 to favor the initial phase of inflammation. **(B)** ARPCs can mediate immunomodulation and affect inflammatory state. The physiological response to tissue damage can be divided into three phases: inflammatory, reparative, and remodeling. During this process, inflammatory status (defined as the types and concentrations of cytokines and cells of the immune system present) changes considerably: proinflammatory influences (red dashed line) are dominant in the inflammatory, infection-fighting phase and diminish in the reparative and remodeling phases that follow, which allows wound healing. In the context of the intensity of the immune response (right vertical axis), the inflammatory response (red dashed line) fluctuates during the wound-healing process. Such changes in inflammation substantially alter the effects of mesenchymal stem cells (MSC)-meditated immunomodulation, which results in a variable correlation between the intensity of inflammation and efficacy of MSC treatment (solid black line). Such changes in inflammation are also affected by ARPC-meditated immunomodulation. ARPCs cause DN T cell decrease at the initial phase, promoting inflammation, and DN T cell increase in the late inflammation stages, favoring the inflammation quenching.

The physiological response to tissue damage usually follows a three-step pattern: inflammatory, reparative, and remodeling. Inflammation status (expressed as the types and quantity of immune system cytokines and cells present) may significantly vary. Inflammation intensity is high in the infection-fighting stage, while decreases in the subsequent reparative and remodeling stages that enable wound healing (63). As a result, ARPCs reduce Tregs and DN T cells in the early stages of a tissue damage, encouraging inflammation, whereas they increase these regulatory cells in the late stages of the tissue repair process, favoring inflammation quenching. In chronic inflammatory event, ARPCs further support DN T cell generation to prevent escalation and related risks (**Figure 2**). The ARPCs therefore reveals useful also in the setting of AKI in which the role of innate immunity in acute tissue injury is well established, with engagement of complement, cytokines, and leucocytes (64).

Different stimuli may modulate the immunomodulatory activity of ARPCs. Both IFN-α or IFN-β can suppress renal progenitor differentiation into mature podocytes (65) whereas no data are present on IFN-γ stimulation on ARPCs. However, tubular epithelial cells express PD-L1, an inducible antigen that negatively regulates T-cell responses elicited by IFN-γ (66). Further studies are needed to deep this recently discovered ARPC ability to regulate T cells.

## Deadalus and Icarus: The Importance of Striking Balance

Still more than two thousand years later, Greek mythology passes an invaluable heritage of knowledge in the form of tales where general concepts can help and lead to a more general level of knowledge. We find in Daedalus and Icarus flight a perfect analogy with the immune balance. Daedalus, to revert his own fate and fly away from their imprisonment, wore wings made of feathers and wax. But the wise father warned him on the importance of a balanced and a fine-tuned control: an unbalanced flight would have eventually resulted in wing consumption by the heat of the sun or the humidity of the sea, leading to fatal loss. Icarus, the boisterous son, disattended the recommendations of his father, paying such uncontrolled, heighten flight with his own life. Such ancient allegoric tale perfectly resembles our modern view of immune system response. Human immune system over- or under-reaction to external or internal stimuli leads to an uncontrolled cascade of effects, eventually leading to death. A closer look to renal progenitor cells in response to an inflammatory environment leads to a close similarity between ARPC and the wise Daedalus, where a tweaked and calibrated regulation in Tregs and DN T cells conveys towards efficient balance between immune tolerance and autoimmunity.

Moving from the awareness that many renal diseases are characterized by inflammatory infiltrating T cells, and the recent identification of prevalence in DN T cells, further research into the role of ARPCs in immune system modulation may pave the road to new therapeutic interventions or adjunct cell-based therapies for both acute and chronic kidney disorders. It is in such direction, that new proposed approaches based on allogenic perinatal stem cells offering an immune privileged locale to the kidney stem/progenitor cells of the recipient can convoy towards innovative therapies, where immune-modulation, rather than immune-suppression, will support regenerative capacity, abetting normal immune response towards eventual infective agents (such as SARS viruses and COVID-19).

Innovative stem cell strategies are promising new cellular tool for advanced medical treatments, currently evaluated and tested by different groups worldwide, aimed to refine and handle balance between immune-repression and immune-stimulation. Recognizing the existence of both suppressive and stimulatory properties, and the mechanisms that underpin the duality of immune reaction, will eventually aid in the development of active immunotherapeutic approaches that manipulate the immune system to achieve therapeutic benefit.

## Data Availability Statement

The original contributions presented in the study are included in the article/supplementary material. Further inquiries can be directed to the corresponding author.

## Author Contributions

FS and RG made the concept and design of the article. AP, CC, KK, and AS drafted the article and contributed to literature search. GC, GBP, and LG critically revised the article. All authors listed have made a substantial, direct, and intellectual contribution to the work and approved it for publication.

## Funding

We thanks PersonGene company and the University of Bari Aldo Moro for supporting this research. The funder was not involved in the study design, collection, analysis, interpretation of data, the writing of this article or the decision to submit it for publication.

## Conflict of Interest

The authors declare that the research was conducted in the absence of any commercial or financial relationships that could be construed as a potential conflict of interest.

## Publisher’s Note

All claims expressed in this article are solely those of the authors and do not necessarily represent those of their affiliated organizations, or those of the publisher, the editors and the reviewers. Any product that may be evaluated in this article, or claim that may be made by its manufacturer, is not guaranteed or endorsed by the publisher.

## References

[1] Charles A Janeway J Travers P Walport M Shlomchik MJ. Principles of Innate and Adaptive Immunity. New York: Garland Science (2001).

[2] Ebert JD. Cellular Immunology. Books 1 and 2. Macfarlane Burnet. Melbourne University Press, Carlton, Victoria, Australia; Cambridge University Press, New York, 1969. X + 728 Pp. + Plates. $18.50. Science (1970) 167:1608–8. doi: 10.1126/science.167.3925.1608-a

[3] Li N Hua J. Interactions Between Mesenchymal Stem Cells and the Immune System. Cell Mol Life Sci (2017) 74:2345–60. doi: 10.1007/s00018-017-2473-5 PMC11107583 28214990

[4] Hossein-khannazer N Torabi S Hosseinzadeh R Shahrokh S Asadzadeh Aghdaei H Memarnejadian A. Novel Cell-Based Therapies in Inflammatory Bowel Diseases: The Established Concept, Promising Results. Hum Cell (2021) 34(5):1289–300. doi: 10.1007/s13577-021-00560-w PMC8165675 34057700

[5] Curci C Picerno A Chaoul N Stasi A de Palma G Franzin R. Adult Renal Stem/Progenitor Cells can Modulate T Regulatory Cells and Double Negative T Cells. Int J Mol Sci (2021) 22:1–16. doi: 10.3390/ijms22010274 PMC7795073 33383950

[6] Wang M Yuan Q Xie L. Mesenchymal Stem Cell-Based Immunomodulation: Properties and Clinical Application. Stem Cells Int (2018) 2018:1–12. doi: 10.1155/2018/3057624 PMC6022321 30013600

[7] Lindau R Vondra S Spreckels J Solders M Svensson-Arvelund J Berg G. Decidual Stromal Cells Support Tolerance at the Human Foetal-Maternal Interface by Inducing Regulatory M2 Macrophages and Regulatory T-Cells. J Reprod Immunol (2021) 146:103330. doi: 10.1016/j.jri.2021.103330 34049032

[8] Wolbank S Peterbauer A Fahrner M Hennerbichler S van Griensven M Stadler G. Dose-Dependent Immunomodulatory Effect of Human Stem Cells From Amniotic Membrane: A Comparison With Human Mesenchymal Stem Cells From Adipose Tissue. Tissue Eng (2007) 13:1173–83. doi: 10.1089/ten.2006.0313 17518752

[9] Liu YH Chan J Vaghjiani V Murthi P Manuelpillai U Toh BH. Human Amniotic Epithelial Cells Suppress Relapse of Corticosteroid-Remitted Experimental Autoimmune Disease. Cytotherapy (2014) 16:535–44. doi: 10.1016/j.jcyt.2013.10.007 24411589

[10] Morandi F Horenstein AL Quarona V Faini AC Castella B Srinivasan RC. Ectonucleotidase Expression on Human Amnion Epithelial Cells: Adenosinergic Pathways and Dichotomic Effects on Immune Effector Cell Populations. J Immunol (2019) 202:724–35. doi: 10.4049/jimmunol.1800432 30587530

[11] Banas R Miller C Guzik L Zeevi A. Amnion-Derived Multipotent Progenitor Cells Inhibit Blood Monocyte Differentiation Into Mature Dendritic Cells. Cell Transplant (2014) 23:1111–25. doi: 10.3727/096368913X670165 23849060

[12] Rizzo R Vercammen M Van De Velde H Horn PA Rebmann V. The Importance of HLA-G Expression in Embryos, Trophoblast Cells, and Embryonic Stem Cells. Cell Mol Life Sci (2011) 68:341–52. doi: 10.1007/s00018-010-0578-1 PMC11114702 21080028

[13] Verloes A Van de Velde H LeMaoult J Mateizel I Cauffman G Horn PA. HLA-G Expression in Human Embryonic Stem Cells and Preimplantation Embryos. J Immunol (2011) 186:2663–71. doi: 10.4049/jimmunol.1001081 21248264

[14] Strom SC Gramignoli R. Human Amnion Epithelial Cells Expressing HLA-G as Novel Cell-Based Treatment for Liver Disease. Hum Immunol (2016) 77:734–9. doi: 10.1016/j.humimm.2016.07.002 27476049

[15] Menier C Rabreau M Challier JC Le Discorde M Carosella ED Rouas-Freiss N. Erythroblasts Secrete the Nonclassical HLA-G Molecule From Primitive to Definitive Hematopoiesis. Blood (2004) 104:3153–60. doi: 10.1182/blood-2004-03-0809 15284117

[16] Crisa L McMaster MT Ishii JK Fisher SJ Salomon DR. Identification of a Thymic Epithelial Cell Subset Sharing Expression of the Class Ib HLA-G Molecule With Fetal Trophoblasts. J Exp Med (1997) 186:289–98. doi: 10.1084/jem.186.2.289 PMC2198976 9221758

[17] Ito T Ito N Saathoff M Stampachiacchiere B Bettermann A Bulfone-Paus S. Immunology of the Human Nail Apparatus: The Nail Matrix is a Site of Relative Immune Privilege. J Invest Dermatol (2005) 125:1139–48. doi: 10.1111/j.0022-202X.2005.23927.x 16354183

[18] Montespan F Deschaseaux F Sensébé L Carosella ED Rouas-Freiss N. Osteodifferentiated Mesenchymal Stem Cells From Bone Marrow and Adipose Tissue Express HLA-G and Display Immunomodulatory Properties in Hla-Mismatched Settings: Implications in Bone Repair Therapy. J Immunol Res (2014) 2014. doi: 10.1155/2014/230346 PMC4022112 24877156

[19] Rebmann V Busemann A Lindemann M Grosse-Wilde H. Detection of HLA-G5 Secreting Cells. In: Human Immunology. Elsevier Inc (2003). p. 1017–24. doi: 10.1016/j.humimm.2003.08.354 14602230

[20] González Á Rebmann V Lemaoult J Horn PA Carosella ED Alegre E. The Immunosuppressive Molecule HLA-G and its Clinical Implications. Crit Rev Clin Lab Sci (2012) 49:63–84. doi: 10.3109/10408363.2012.677947 22537084

[21] Amiot L Vu N Samson M. Biology of the Immunomodulatory Molecule HLA-G in Human Liver Diseases. J Hepatol (2015) 62:1430–7. doi: 10.1016/j.jhep.2015.03.007 25772038

[22] Cai YJ Huang L Leung TY Burd A. A Study of the Immune Properties of Human Umbilical Cord Lining Epithelial Cells. Cytotherapy (2014) 16:631–9. doi: 10.1016/j.jcyt.2013.10.008 24364910

[23] Morandi F Marimpietri D Görgens A Gallo A Srinivasan RC El-Andaloussi S. Human Amnion Epithelial Cells Impair T Cell Proliferation: The Role of HLA-G and HLA-E Molecules. Cells (2020) 9. doi: 10.3390/cells9092123 PMC7563681 32961693

[24] González-Hernandez A LeMaoult J Lopez A Alegre E Caumartin J Le Rond S. Linking Two Immuno-Suppressive Molecules: Indoleamine 2,3 Dioxygenase can Modify HLA-G Cell-Surface Expression. Biol Reprod (2005) 73:571–8. doi: 10.1095/biolreprod.105.040089 15878889

[25] Moreau P Adrian-Cabestre F Menier C Guiard V Gourand L Dausset J. IL-10 Selectively Induces HLA-G Expression in Human Trophoblasts and Monocytes. Int Immunol (1999) 11:803–11. doi: 10.1093/intimm/11.5.803 10330285

[26] Kolanko E Kopaczka K Koryciak-Komarska H Czech E Szmytkowska P Gramignoli R. Increased Immunomodulatory Capacity of Human Amniotic Cells After Activation by Pro-Inflammatory Chemokines. Eur J Pharmacol (2019) 859. doi: 10.1016/j.ejphar.2019.172545 31319066

[27] Yang Y Geraghty DE Hunt JS. Cytokine Regulation of HLA-G Expression in Human Trophoblast Cell Lines. J Reprod Immunol (1995) 29:179–95. doi: 10.1016/0165-0378(95)00942-E 8636924

[28] Mouillot G Marcou C Zidi I Guillard C Sangrouber D Carosella ED. Hypoxia Modulates HLA-G Gene Expression in Tumor Cells. Hum Immunol (2007) 68:277–85. doi: 10.1016/j.humimm.2006.10.016 17400064

[29] Dominici M Le Blanc K Mueller I Slaper-Cortenbach I Marini FC Krause DS. Minimal Criteria for Defining Multipotent Mesenchymal Stromal Cells. The International Society for Cellular Therapy Position Statement. Cytotherapy (2006) 8:315–7. doi: 10.1080/14653240600855905 16923606

[30] Sträter N. Ecto-5′-Nucleotidase: Structure Function Relationships. Purinergic Signalling (2006) 2:343–50. doi: 10.1007/s11302-006-9000-8 PMC2254484 18404474

[31] Horenstein AL Chillemi A Zaccarello G Bruzzone S Quarona V Zito A. A CD38/CD203A/CD73 Ectoenzymatic Pathway Independent of CD39 Drives a Novel Adenosinergic Loop in Human T Lymphocytes. OncoImmunology (2013) 2. doi: 10.4161/onci.26246 PMC3850273 24319640

[32] Grahnert A Grahnert A Klein C Schilling E Wehrhahn J Hauschildt S. NAD +: A Modulator of Immune Functions. Innate Immun (2011) 17:212–23. doi: 10.1177/1753425910361989 20388721

[33] Morandi F Horenstein AL Chillemi A Quarona V Chiesa S Imperatori A. CD56 Bright CD16 – NK Cells Produce Adenosine Through a CD38-Mediated Pathway and Act as Regulatory Cells Inhibiting Autologous CD4 + T Cell Proliferation. J Immunol (2015) 195:965–72. doi: 10.4049/jimmunol.1500591 26091716

[34] Morandi F Morandi B Horenstein AL Chillemi A Quarona V Zaccarello G. A non-Canonical Adenosinergic Pathway Led by CD38 in Human Melanoma Cells Induces Suppression of T Cell Proliferation. Oncotarget (2015) 6:25602–18. doi: 10.18632/oncotarget.4693 PMC4694853 26329660

[35] Gramignoli R. Therapeutic Use of Human Amnion-Derived Products: Cell-Based Therapy for Liver Disease. Curr Pathobiol Rep (2016) 4:157–67. doi: 10.1007/s40139-016-0112-8

[36] Uckan D. Trophoblasts Express Fas Ligand: A Proposed Mechanism for Immune Privilege in Placenta and Maternal Invasion. Mol Hum Reprod (1997) 3:655–62. doi: 10.1093/molehr/3.8.655 9294848

[37] Tena A Sachs DH. Stem Cells: Immunology and Immunomodulation. Cell-Based Ther Retinal Degenerative Dis (2014) 53:122–32. doi: 10.1159/000357360 24732766

[38] Magatti M Vertua E Cargnoni A Silini A Parolini O. The Immunomodulatory Properties of Amniotic Cells: The Two Sides of the Coin. Cell Transplant (2018) 27:31–44. doi: 10.1177/0963689717742819 29562786 PMC6434482

[39] Angelotti ML Ronconi E Ballerini L Peired A Mazzinghi B Sagrinati C. Characterization of Renal Progenitors Committed Toward Tubular Lineage and Their Regenerative Potential in Renal Tubular Injury. Stem Cells (2012) 30:1714–25. doi: 10.1002/stem.1130 22628275

[40] Sciancalepore AG Portone A Moffa M Persano L De Luca M Paiano A. Micropatterning Control of Tubular Commitment in Human Adult Renal Stem Cells. Biomaterials (2016) 94:57–69. doi: 10.1016/j.biomaterials.2016.03.042 27105437

[41] Sciancalepore AG Sallustio F Girardo S Gioia Passione L Camposeo A Mele E. Pisignano D. A Bioartificial Renal Tubule Device Embedding Human Renal Stem/Progenitor Cells. PloS One (2014) 9:e87496. doi: 10.1371/journal.pone.0087496 24498117 PMC3907467

[42] Bussolati B Tetta C Camussi G. Contribution of Stem Cells to Kidney Repair. Am J Nephrol (2008) 28:813–22. doi: 10.1159/000137681 18535367

[43] Sallustio F De Benedictis L Castellano G Zaza G Loverre A Costantino V. TLR2 Plays a Role in the Activation of Human Resident Renal Stem/Progenitor Cells. FASEB J (2010) 24:514–25. doi: 10.1096/fj.09-136481 19843711

[44] Sallustio F Serino G Schena FP. Potential Reparative Role of Resident Adult Renal Stem/Progenitor Cells in Acute Kidney Injury. Biores Open Access (2015) 4:326–33. doi: 10.1089/biores.2015.0011 PMC4509615 26309808

[45] Sciancalepore AG Sallustio F Girardo S Passione LG Camposeo A Mele E. Correction: A Bioartificial Renal Tubule Device Embedding Human Renal Stem/Progenitor Cells. PloS One (2015) 10:e0128261. doi: 10.1371/journal.pone.0128261 25955358 PMC4425656

[46] Sallustio F Curci C Aloisi A Toma CC Marulli E Serino G. Inhibin-A and Decorin Secreted by Human Adult Renal Stem/Progenitor Cells Through the TLR2 Engagement Induce Renal Tubular Cell Regeneration. Sci Rep (2017) 7:8225. doi: 10.1038/s41598-017-08474-0 28811645 PMC5557965

[47] Romagnani P Lasagni L Remuzzi G. Renal Progenitors: An Evolutionary Conserved Strategy for Kidney Regeneration. Nat Rev Nephrol (2013) 9:137–46. doi: 10.1038/nrneph.2012.290 23338209

[48] Sallustio F Serino G Costantino V Curci C Cox SN De Palma G. miR-1915 and miR-1225-5p Regulate the Expression of CD133, PAX2 and TLR2 in Adult Renal Progenitor Cells. PloS One (2013) 8. doi: 10.1371/journal.pone.0068296 PMC3704645 23861881

[49] Sallustio F Gesualdo L Pisignano D. The Heterogeneity of Renal Stem Cells and Their Interaction With Bio- and Nano-Materials. In: Advances in Experimental Medicine and Biology. Springer New York LLC (2019). p. 195–216. doi: 10.1007/978-3-030-11096-3_12 31016602

[50] Romoli S Angelotti ML Antonelli G Kumar VRS Mulay SR Desai J. CXCL12 Blockade Preferentially Regenerates Lost Podocytes in Cortical Nephrons by Targeting an Intrinsic Podocyte-Progenitor Feedback Mechanism. Kidney Int (2018) 94:1111–26. doi: 10.1016/j.kint.2018.08.013 PMC6251974 30385042

[51] Lazzeri E Angelotti ML Peired A Conte C Marschner JA Maggi L. Endocycle-Related Tubular Cell Hypertrophy and Progenitor Proliferation Recover Renal Function After Acute Kidney Injury. Nat Commun (2018) 9:1344. doi: 10.1038/s41467-018-03753-4 29632300 PMC5890293

[52] Gramignoli R Sallustio F Widera D Raschzok N. Editorial: Tissue Repair and Regenerative Mechanisms by Stem/Progenitor Cells and Their Secretome. Front Med (2019) 6:11. doi: 10.3389/fmed.2019.00011 PMC6365979 30766874

[53] Sallustio F Curci C Stasi A De Palma G Divella C Gramignoli R. Role of Toll-Like Receptors in Actuating Stem/Progenitor Cell Repair Mechanisms: Different Functions in Different Cells. Stem Cells Int (2019) 2019. doi: 10.1155/2019/6795845 PMC6476106 31089331

[54] Sallustio F Stasi A Curci C Divella C Picerno A Franzin R. Renal Progenitor Cells Revert LPS-Induced Endothelial-to-Mesenchymal Transition by Secreting CXCL6, SAA4, and BPIFA2 Antiseptic Peptides. FASEB J (2019) 33:10753–66. doi: 10.1096/fj.201900351R 31268775

[55] Seo HS Michalek SM Nahm MH. Lipoteichoic Acid is Important in Innate Immune Responses to Gram-Positive Bacteria. Infection Immun (2008) 76:206–13. doi: 10.1128/IAI.01140-07 PMC2223632 17954723

[56] Ranganath SH Levy O Inamdar MS Karp JM. Harnessing the Mesenchymal Stem Cell Secretome for the Treatment of Cardiovascular Disease. Cell Stem Cell (2012) 10:244–58. doi: 10.1016/j.stem.2012.02.005 PMC3294273 22385653

[57] Deng Y Zhang Y Ye L Zhang T Cheng J Chen G. Umbilical Cord-Derived Mesenchymal Stem Cells Instruct Monocytes Towards an IL10-Producing Phenotype by Secreting IL6 and HGF. Sci Rep (2016) 6. doi: 10.1038/srep37566 PMC5137158 27917866

[58] Luz-Crawford P Kurte M Bravo-Alegría J Contreras R Nova-Lamperti E Tejedor G. Mesenchymal Stem Cells Generate a CD4+CD25+Foxp3+ Regulatory T Cell Population During the Differentiation Process of Th1 and Th17 Cells. Stem Cell Res Ther (2013) 4:65. doi: 10.1186/scrt216 23734780 PMC3706898

[59] Martina MN Noel S Saxena A Bandapalle S Majithia R Jie C. Double-Negative αβ T Cells Are Early Responders to AKI and Are Found in Human Kidney. J Am Soc Nephrol : JASN (2016) 27:1113–23. doi: 10.1681/ASN.2014121214 PMC4814175 26315532

[60] Juvet SC Zhang L. Double Negative Regulatory T Cells in Transplantation and Autoimmunity: Recent Progress and Future Directions. J Mol Cell Biol (2012) 4:48–58. doi: 10.1093/jmcb/mjr043 22294241 PMC3269300

[61] D’Acquisto F Crompton T. CD3+CD4–CD8– (Double Negative) T Cells: Saviours or Villains of the Immune Response? Biochem Pharmacol (2011) 82:333–40. doi: 10.1016/j.bcp.2011.05.019 21640713

[62] Chen W Ford MS Young KJ Zhang L. The Role and Mechanisms of Double Negative Regulatory T Cells in the Suppression of Immune Responses. Cell Mol Immunol (2004) 1:328–35. 16285891

[63] Wang Y Chen X Cao W Shi Y. Plasticity of Mesenchymal Stem Cells in Immunomodulation: Pathological and Therapeutic Implications. Nat Immunol (2014) 15:1009–16. doi: 10.1038/ni.3002 25329189

[64] Rabb H. Immune Modulation of Acute Kidney Injury. J Am Soc Nephrol : JASN (2006) 17:604–6. doi: 10.1681/ASN.2006010060 16481410

[65] Migliorini A Angelotti ML Mulay SR Kulkarni OO Demleitner J Dietrich A. The Antiviral Cytokines IFN-α and IFN-β Modulate Parietal Epithelial Cells and Promote Podocyte Loss: Implications for IFN Toxicity, Viral Glomerulonephritis, and Glomerular Regeneration. Am J Pathol (2013) 183:431–40. doi: 10.1016/j.ajpath.2013.04.017 23747509

[66] Schoop R Wahl P le Hir M Heemann U Wang M Wüthrich RP. Suppressed T-Cell Activation by IFN- -Induced Expression of PD-L1 on Renal Tubular Epithelial Cells. Nephrol Dialysis Transplant (2004) 19:2713–20. doi: 10.1093/ndt/gfh423 15353579

